# Anomalous High Rainfall and Soil Saturation as Combined Risk Indicator of Rift Valley Fever Outbreaks, South Africa, 2008–2011

**DOI:** 10.3201/eid2212.151352

**Published:** 2016-12

**Authors:** Roy Williams, Johan Malherbe, Harold Weepener, Phelix Majiwa, Robert Swanepoel

**Affiliations:** Agricultural Research Council–Onderstepoort Veterinary Institute, Onderstepoort, South Africa (R. Williams, P. Majiwa);; Agricultural Research Council–Institute for Soil, Climate and Water, Pretoria, South Africa (J. Malherbe, H. Weepener);; University of Pretoria, Onderstepoort (R. Swanepoel)

**Keywords:** Rift Valley fever, forecasting, soil saturation, rainfall, vector-borne infections, South Africa, outbreaks, epizootics, epidemics, risk indicator, zoonoses, prediction models, viruses, livestock, vaccination, herd immunity, mosquito vectors, mosquitoes

## Abstract

Rift Valley fever (RVF), a zoonotic vectorborne viral disease, causes loss of life among humans and livestock and an adverse effect on the economy of affected countries. Vaccination is the most effective way to protect livestock; however, during protracted interepidemic periods, farmers discontinue vaccination, which leads to loss of herd immunity and heavy losses of livestock when subsequent outbreaks occur. Retrospective analysis of the 2008–2011 RVF epidemics in South Africa revealed a pattern of continuous and widespread seasonal rainfall causing substantial soil saturation followed by explicit rainfall events that flooded dambos (seasonally flooded depressions), triggering outbreaks of disease. Incorporation of rainfall and soil saturation data into a prediction model for major outbreaks of RVF resulted in the correctly identified risk in nearly 90% of instances at least 1 month before outbreaks occurred; all indications are that irrigation is of major importance in the remaining 10% of outbreaks.

## Abstract 2

A prediction model that includes these factors shows promising potential for forecasting major outbreaks.

Rift Valley fever (RVF) is an acute viral disease of livestock and humans in Africa, Madagascar, the Comoros Archipelago, and the Arabian Peninsula. Infection is caused by RVF virus (family *Bunyaviridae*, genus *Phlebovirus*), a zoonotic mosquitoborne virus. In animals, RVF causes abortion in pregnant sheep, goats, cattle, and camels, and it can cause death, particularly in newborn animals. Humans become infected through contact with the tissues of infected animals or, less commonly, from the bite of infected mosquitoes; infection usually results in a benign febrile illness, although complications, such as ocular sequelae or fatal encephalitis and hemorrhagic disease, can occur (*1*,*2*). Large epidemics occur at irregular intervals of 5–15 years, or longer, when heavy rainfall facilitates the breeding of the mosquito vectors, and result in substantial economic losses due to livestock deaths and restrictions on animal trade (*3*).

RVF was discovered in Kenya in 1930 and was subsequently found in many countries of sub-Saharan Africa (*1*); spread beyond this region into Egypt, Saudi Arabia, and Yemen was noted from 1977 to 2007 (*4*). The disease was first reported in South Africa during 1950–1951, when a large epidemic occurred in the country’s central plateau; 2 additional major epidemics affected the same area in 1974–1976 and 2010–2011 and extended into neighboring provinces (*5*,*6*). Limited outbreaks were recorded during the intervening years; these outbreaks initially occurred on the central plateau, but starting in the 1980s, they increasingly occurred in northeastern parts of the country. Outbreaks among animals are defined as the occurrence of >1 confirmed cases within an epidemiologic unit, meaning a group of animals that share approximately the same likelihood of exposure to infection (*7*). In the absence of nomadism in South Africa, epidemiologic units in the country essentially coincide with geographic locations of commercial farms or communal grazing areas. Epidemics are not defined, but the term is applied arbitrarily to the occurrence of intense or multiple outbreaks in >1 epidemiologic units.

A phylogenetic study of RVF isolates (*8*) indicated that 2 different virus lineages, C and H, were responsible for the 2008–2011epidemics in South Africa. The 2008 and 2009 epidemics in the northeast were associated with lineage C virus and were much less intense than the subsequent epidemic associated with lineage H virus in the central plateau in 2010–2011 (*6*,*8*). However, lineage C virus was associated with major epidemics in Zimbabwe, eastern Africa, Saudi Arabia and Yemen. Furthermore, in 2004, lineage H was associated with only 1 human infection in Namibia in the absence of reported disease in livestock. Thus, differences in epidemic intensity were more likely determined by epidemiologic factors (e.g., climate, topography, vegetation) or vector species than by the virus strains involved.

Distinction is made between 2 types of RVF virus vectors. Floodwater-breeding *Aedes* mosquitoes of the subgenera *Aedimorphus* and *Neomelaniconion* are regarded as endemic or maintenance vectors because they are thought to be responsible for ensuring long-term survival of the virus through transovarial transmission of infection. Their feeding and egg-laying cycle are completed within 3 weeks of hatching; eggs are laid in mud at the edges of rainwater that temporarily accumulates in pans (shallow depressions), vleis (seasonal wetlands), and the banks of dams and watercourses, collectively known as dambos in Africa. Not all sites flood directly as a result of local precipitation; river banks, large dams, and irrigation schemes may flood weeks to months after heavy rains occur in remote catchment areas (*9*). In contrast to other mosquitoes, the eggs of floodwater-breeding aedines require conditioning by a period of partial desiccation as the water level recedes before they will hatch once they get wet again during the next flood period. Thus, they overwinter as eggs, which can survive for long periods in dried mud, possibly for several seasons if dambos remain dry (*10*). After adequate rainfall floods breeding sites, infected aedines emerge and transmit the RVF virus to available susceptible animals that serve as amplifying hosts for transmission of infection to the principal epidemic vectors, mainly *Culex* species mosquitoes. Other epidemic vectors include other culicines, anophelines, and even biting flies that act as mechanical vectors. Epidemic mosquito vectors breed on standing bodies of water and are able to sustain and spread outbreaks (*11*,*12*).

In RVF virus–endemic areas with warm and moist climates, infected aedines can emerge each year, and even infected culicines can hibernate as adults, resulting in regular exposure of livestock to RVF virus. Thus, most animals are immune by breeding age and are able to transfer maternal immunity to their offspring; hence, disease is seldom seen (*1*). In more arid areas, particularly those with cold winters and prolonged dry spells (e.g., the central plateau of South Africa), intervals between outbreaks may extend to decades. During these long intervals, livestock populations are replaced by animals susceptible to RVF virus, and heavy rains can trigger major epidemics among such animals.

Because it is difficult to convince livestock owners and veterinary authorities to vaccinate livestock during protracted interepidemic periods, attempts have been made to provide early warning of impending outbreaks through remote sensing of climate patterns conducive to large-scale emergence of vectors (*13*,*14*). Measurement of vegetation photosynthetic activity through satellite imaging is used to derive a normalized difference vegetation index (NDVI) as a surrogate for precipitation. NDVI anomalies that exceed the long-term mean (LTM) are interpreted as favorable for the occurrence of outbreaks (*14*), although a lag of 2–8 weeks between heavy rains and the subsequent increase in the NDVI (*15*,*16*) reduces its value for risk mitigation. The ENSO (El Niño and Southern Oscillation) phenomenon is a major determinant of global interannual climate variability, and anomalies, expressed in terms of a derived Southern Oscillation Index (SOI), are used to predict the occurrence of abnormal rainfall with a lead time of 2–5 months; positive SOI (La Niña) events usually precede heavy rains in southern Africa (*17*,*18*). However, such regional forecasts perform poorly for specific geographic locations; thus, SOI can be considered only as a supplementary preseason indicator for the risk of RVF outbreaks. In contrast, soil moisture status is a reliable indicator of potential flash floods on small catchments (*19*) and therefore has potential to indicate when ground is sufficiently saturated for dambos to be flooded after heavy rains. The combined effect of soil saturation and precipitation could therefore serve as a potential risk indicator of optimal ecoclimatic conditions for the upsurge of mosquito vector populations and subsequent outbreaks of RVF. We evaluated the combined aspects of anomalous high rainfall and concurrent soil saturation in a RVF forecast model for South Africa, using accurate temporal and spatial records of the 2008–2011 epidemics to derive risk maps (Directorate of Animal Health, Department of Agriculture, Forestry and Fisheries, pers. comm., 2012 Jul 6).

## Data and Methods

### RVF Base Map

We constructed an interpolated base map of 1-km spatial resolution, representing the probability of risk for RVF outbreaks throughout South Africa, by using ArcGIS version 10.2.2 (ESRI, Redlands, CA, USA). Interpolation data points included locations of all historic sites of RVF outbreaks from 1950 to 2011 (Directorate of Animal Health, Department of Agriculture, Forestry and Fisheries, pers. comm., 2012 Jul 6) (*6*); additional data points were placed along the perimeters of buffer zones at radii of 30, 50, and 90 km around historic sites. Historic sites were allocated an interpolation value of 1.0, and the values of additional data points were based on the distance from the nearest historic site, decreasing incrementally by a factor of 0.1 for every 10 km; probability values ranged from 1.0 to 0.1. Potential risk areas were empirically grouped into 3 classes based on the distance from historic sites: high risk (<20 km from historic sites), moderate risk (>20 km to <40 km), and low risk (>40 km).

### Rainfall and NDVI

The rainfall dataset was produced in near real time (*20*) from a combination of weather station data from the Agricultural Research Council–Institute for Soil, Climate and Water (http://www.arc.agric.za/Pages/Home.aspx) and from the South African Weather Service (http://www.weathersa.co.za/). Weather station data were combined with satellite rainfall estimates available through the Africa Data Dissemination Service for the Famine Early Warning System Network project (http://www.fews.net/). Monthly LTM rainfall was computed in 1-km spatial resolution from monthly rainfall data for 1985–2011 and used to determine the maximum of monthly LTMs during this period. Anomalies of monthly rainfall during January 2007–May 2012 were computed as the percentage deviation from the maximum LTM: monthly rainfall anomaly = (monthly rainfall − maximum LTM) × 100/maximum LTM.

SPOT NDVI data were downloaded through the VEGETATION Program (http://www.spot-vegetation.com/) and the VGT4AFRICA project (http://postel.obs-mip.fr/?VGT4AFRICA-Project,147), developed by the European Commission and disseminated in Africa through GEONETCast (http://wiki.geonetcast.org/geonetcast/html/index.php/Main_Page). Using the method described for rainfall, we computed anomalies of monthly NDVI for January 2007–May 2012 as the percentage deviation from the maximum LTM.

### Soil Saturation Index

The soil saturation index (SSI) represents automated real-time computations of the TOPKAPI hydrologic model, which was adapted to run continuously as a collection of independent 1-km cells at 3-hour intervals (*19*,*21*) beginning in August 2008 (University of KwaZulu-Natal, School of Civil Engineering, Surveying and Construction Management, pers. comm., 2013 Feb 10). Monthly SSI anomalies were computed as the percentage deviation from the LTM (2008–2013) and then computed as 3-month rolling mean SSI anomalies (*14*) for each month from August 2008 through May 2012. For functional compatibility with other model components, SSI anomalies were reclassified as follows: values of 0–3% were reclassified to a value of 0.1, values >3%–6% were reclassified to 0.2, values >6%–9% were reclassified to 0.3, values >9%–12% were reclassified to 0.5, values >12%–15% were reclassified to 0.7, values >15%–18% were reclassified to 0.8, values >18%–20% were reclassified to 0.9, and values >20% were reclassified to 1.0.

### Risk Forecast Model

The aim of the forecast model was to map areas at risk for RVF outbreaks based on the combined effect of anomalous high rainfall and soil saturation but regulated by the risk probability as defined by the base map. Risk was computed as follows: risk = monthly rainfall anomaly × 3-month rolling mean SSI anomaly × base map. Risk maps for January–July 2008 were computed without SSI data. Three-month rolling maximum risk maps were used to reflect changing conditions of mosquito habitats (*14*) during January 2007–June 2011. Pixel values were empirically classified as low risk (<0%), moderate risk (0%–50%), and high risk (>50%).

### Retrospective Evaluation

The accuracy of the model was evaluated by extracting the risk values of all recorded 2008–2011 outbreaks from their relevant risk maps, and we tabled the results according to the month of outbreak and sorted into 1 of the 3 risk classes. Outbreaks in moderate or high risk areas were considered as correctly identified, and outbreaks in low risk areas were considered as incorrectly identified.

## Results

### Rift Valley Fever Base Map

**Figure 1:**
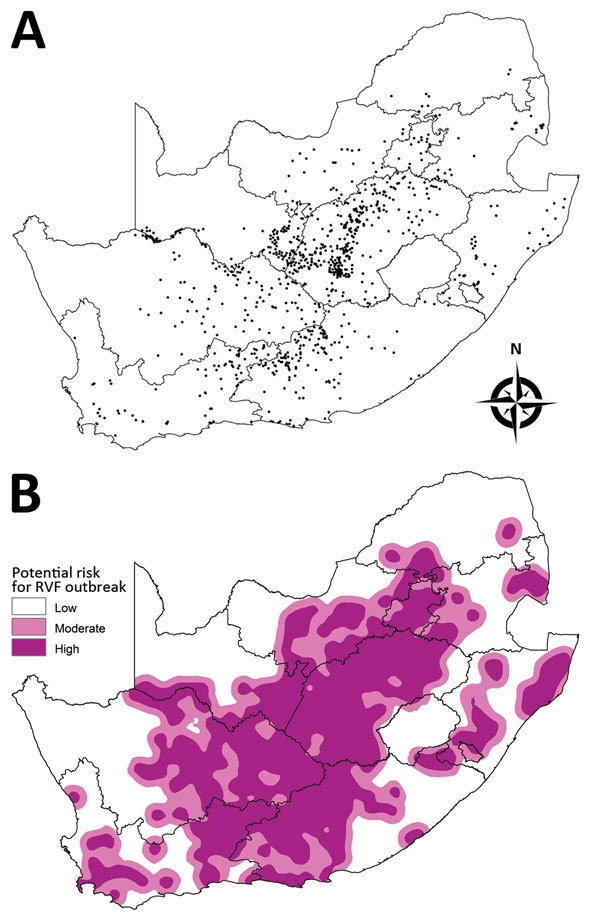
Historic sites of Rift Valley fever (RVF) outbreaks in South Africa from 1950 through 2011 (A) and a base map indicating areas at low, moderate, and high risk for an outbreak (B). Each dot in panel A represents a RVF outbreak. The base map in panel B was created by an interpolation method based on the distance from historic sites: high risk (<20 km), moderate risk (>20 km to <40 km), and low risk (>40 km).

All historic sites of RVF outbreaks in South Africa from 1950 through 2011 were mapped (Figure 1, panel A). The base map (Figure 1, panel B) represents the probability of risk for RVF outbreaks; this probability decreases as the distance from outbreak sites increases.

### Regions of Outbreaks

**Figure 2:**
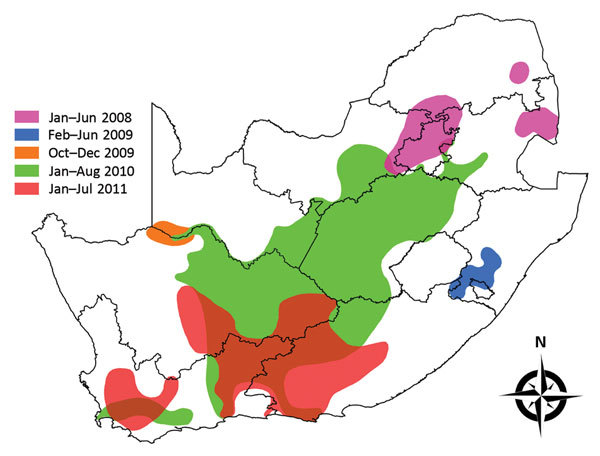
Five regions in South Africa where Rift Valley fever outbreaks occurred during the epidemics of 2008–2011. Regions are grouped, by color, according to their temporal history of outbreaks.

Outbreaks during the epidemics of 2008–2011 were grouped by temporal history into 5 geographic regions (Figure 2). The periods and regions were 1) January–June 2008, Mpumalanga Province and adjacent parts of Limpopo, Gauteng, and North West Provinces; 2) February–June 2009, southern KwaZulu-Natal Province; 3) October–November 2009, Orange River region in Northern Cape Province; 4) January–August 2010, central plateau, including Free State Province and adjacent parts of North West and Northern, Eastern, and Western Cape Provinces; and 5) January–July 2011, adjacent parts of the inland region of Northern, Eastern, and Western Cape Provinces.

### Rainfall, SSI and NDVI

**Figure 3:**
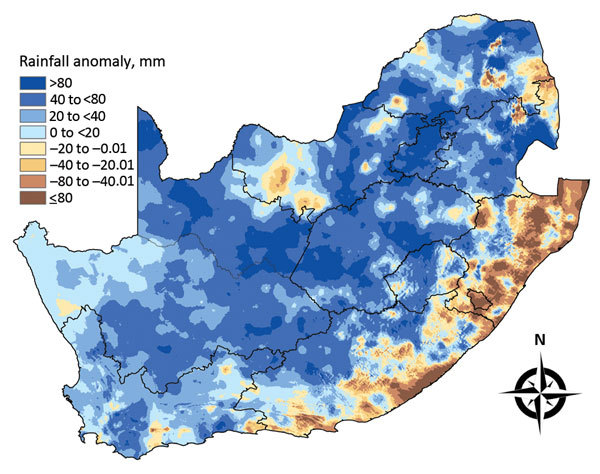
Mean seasonal rainfall anomalies for 4 consecutive seasons (November–March) in South Africa, 2007–2011. The anomalies were computed as deviations from the seasonal long-term mean for 1985–2011.

**Figure 4:**
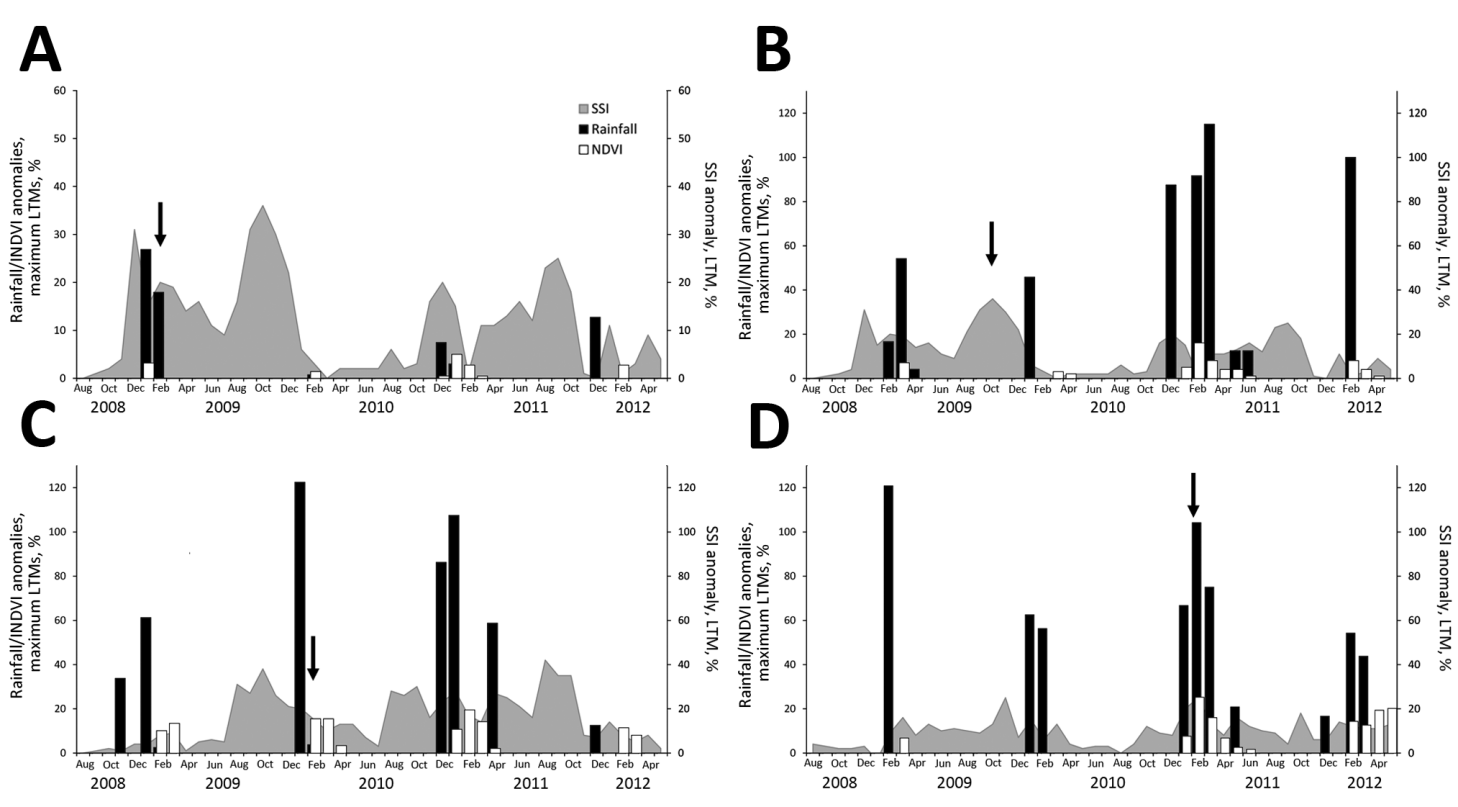
Comparison of monthly rainfall amounts, normalized difference vegetation indices (NDVIs), and soil saturation indices (SSIs) for August 2008–May 2012 for the following 4 areas of South Africa where Rift Valley fever epidemics occurred: A) Southern region of KwaZulu-Natal Province (outbreaks in February–March 2009); B) Orange River region in Northern Cape Province (outbreaks in October–November 2009); C) Bultfontein area of Free State Province (outbreaks in January–February 2010); D) Graaff-Reinet area of Eastern Cape Province (outbreaks in January–February 2011). Rainfall and NDVI anomalies were computed as percentage of the maximum of the long-term means (LTMs); SSI anomalies were computed as percentage of the LTM. Arrows indicate time of first outbreak in the region.

Rainfall data for 2008–2011 indicated a pattern of incessant and widespread seasonal rainfall (Figure 3), resulting in substantial soil saturation, after which explicit rainfall events triggered subsequent outbreaks of RVF in different regions. We also compared rainfall, NDVI, and monthly SSI data from August 2008 through May 2012 for outbreaks that occurred in the first 2 months of each of the following RVF epidemics: 1) southern KwaZulu-Natal Province (February–March 2009), 2) Orange River region in Northern Cape Province (October–November 2009), 3) Bultfontein area of Free State Province (January–February 2010), and 4) Graaff-Reinet area of Eastern Cape Province (January–February 2011) (Figure 4). No SSI data were available for outbreaks that occurred in Mpumalanga Province and adjacent parts of Limpopo, Gauteng, and North West Provinces during January–June 2008.

In the Bultfontein area of Free State Province, where the major inland epidemic of 2010 began (Figure 4, panel C), 2 major rainfall events in November 2008 and January 2009 with concurrent SSI anomalies <5% did not initiate any RVF outbreaks. However, in January 2010, outbreaks started to occur in the region after at least 4 successive months of SSI anomalies above 20% and a major rainfall event in December 2009. Similarly, outbreaks in southern KwaZulu-Natal Province (Figure 4, panel A) and outbreaks that started in the Graaff-Reinet area of Eastern Cape Province (Figure 4, panel D) occurred after rainfall events that were preceded by 1 month of SSI anomalies above 20%. Anomalous rainfall events were regularly followed by elevated NDVI anomalies (Figure 4, panels A, B, and D), usually after a lag of 2–8 weeks. The outbreaks in the Orange River region of Northern Cape Province did not show the same pattern as the previous 3 instances (Figure 4, panel B). Although outbreaks were preceded by 2 months of SSI anomalies >20%, no major rainfall event occurred before the outbreaks, and no elevated NDVI anomalies occurred concurrently with the outbreaks in this region. This finding suggests that irrigation, which is used in vineyards and orchards along the river in this region, could have been responsible for these outbreaks.

Outbreaks of the epidemics of 2010 and 2011 showed a degree of spatial overlap (Figure 2); no outbreaks occurred in Free State Province in 2011, despite highly suitable climatic conditions throughout the season (Figure 4, panel C). A similar pattern was seen for human RVF infections in 2011, when human cases primarily occurred in areas south of the Orange River, away from Free State Province (*22*). The lack of outbreaks in livestock and humans in Free State Province in 2011 was attributed to accumulated herd immunity, which was believed to be the combined result of natural infections in and vaccination of livestock in the province (*22*).

### RVF Risk Maps

#### January–June 2008 Outbreaks

**Figure 5:**
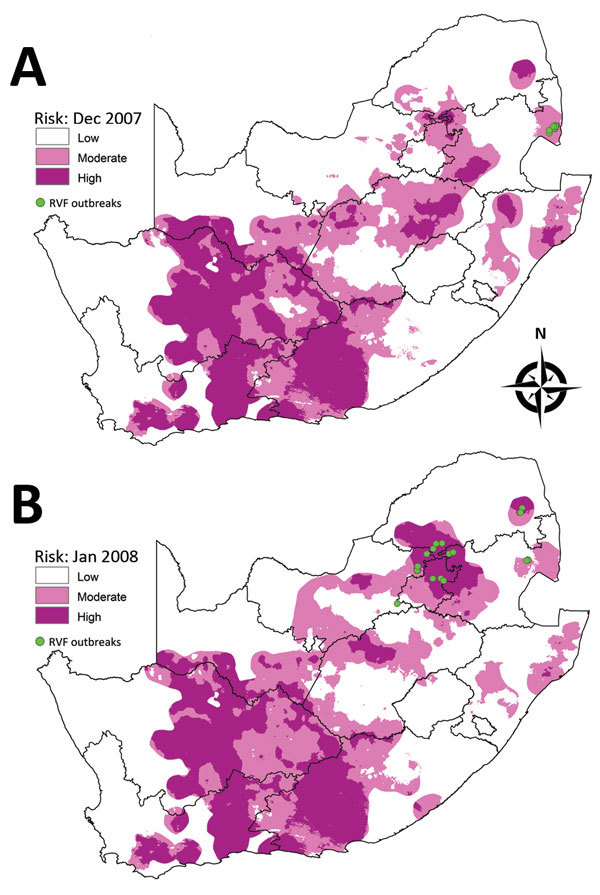
Risk maps for probability of Rift Valley fever (RVF) outbreaks in different areas of South Africa. A) Map for December 2007 showing subsequent outbreaks in January and February 2008. B) Map for January 2008 showing subsequent outbreaks during March–June 2008.

The first outbreaks of the 2008 epidemic in South Africa were recorded in the northeastern part of the country in the region of Kruger National Park; 17 outbreaks were recorded in the area during January–March 2008, and 4 were recorded in June. During March–May 2008, a total of 14 outbreaks were recorded in Gauteng Province and adjacent parts of Limpopo and North West Provinces. The risk map for December 2007 showed moderate risk for the area where outbreaks occurred in January and February 2008 (Figure 5, panel A), and the risk map for January 2008 indicated high to moderate risk in the regions where outbreaks occurred during March–June 2008 (Figure 5, panel B).

#### February–June 2009 Outbreaks

**Figure 6:**
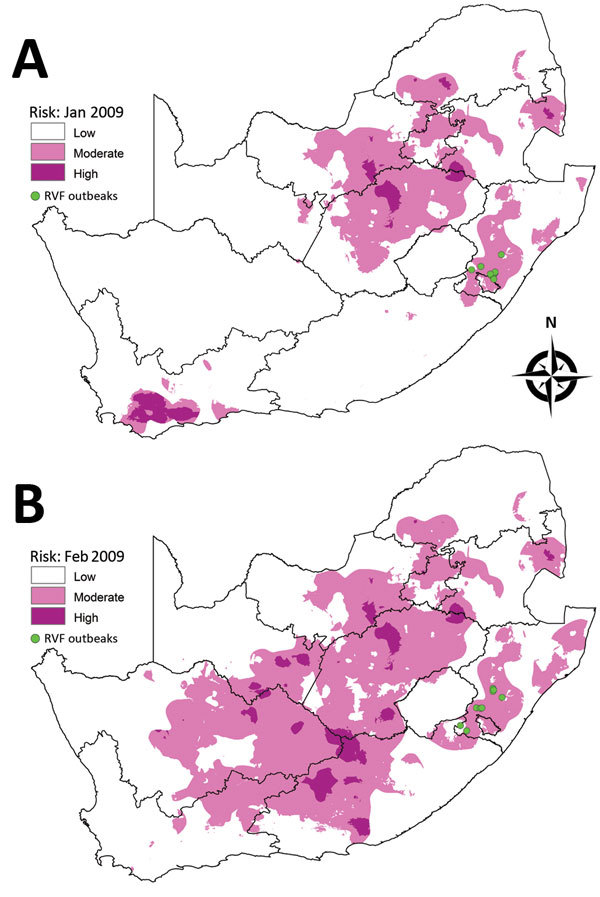
Risk maps for probability of Rift Valley fever (RVF) outbreaks in different areas of South Africa. A) Map for January 2009 showing subsequent outbreaks in February and March 2009. B) Map for February 2009 showing subsequent outbreaks during April–June 2009.

Outbreaks in the southern region of KwaZulu-Natal Province started in February 2009 with 6 outbreaks, followed by 4 outbreaks in March and another 9 during April–June. Areas where outbreaks occurred in February and March 2009 were shown as moderate risk in the risk map for January (Figure 6, panel A); likewise, areas where outbreaks occurred during April–June 2009 were shown as moderate risk in the risk map for February (Figure 6, panel B).

#### October–December 2009 Outbreaks

**Figure 7:**
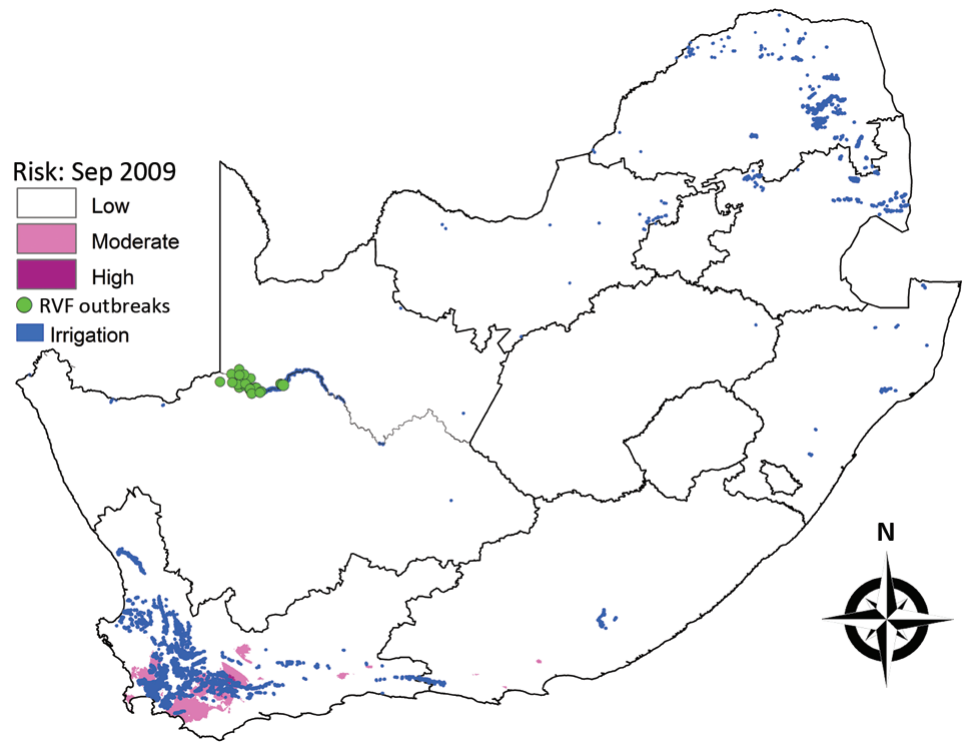
Risk map for probability of Rift Valley fever (RVF) outbreaks in different areas of South Africa. Map for September 2009 indicates irrigation areas and subsequent outbreaks during October–December 2009.

A total of 38 outbreaks occurred during October–December 2009 in the Orange River area of Northern Cape Province, close to the border with Namibia, although the risk map for September 2009 did not indicate any risk in this region (Figure 7). The outbreaks did, however, coincided with irrigation activity along the river. 

#### January–August 2010 Outbreaks

**Figure 8:**
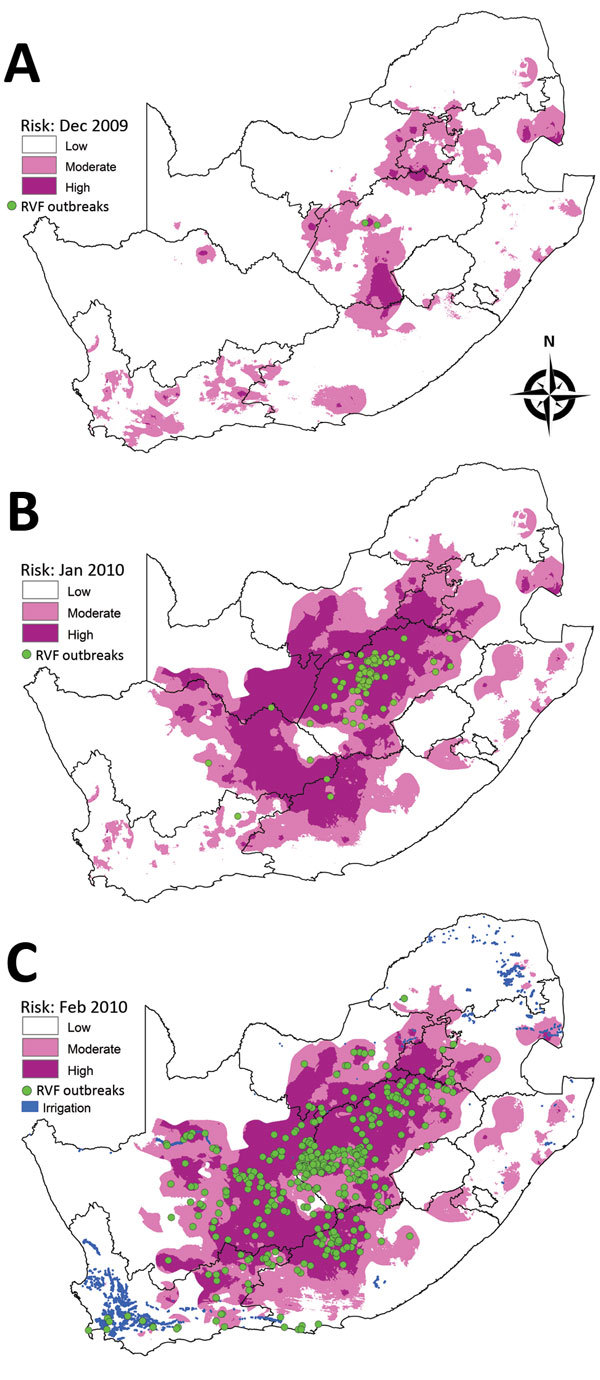
Risk maps for probability of Rift Valley fever (RVF) outbreaks in different areas of South Africa. A) Map for December 2009 showing subsequent outbreaks in January 2010. B) Map for January 2010 showing subsequent outbreaks in February 2010. C) Map for February 2010 indicating irrigation areas and subsequent outbreaks during March–June 2010.

In January 2010, two outbreaks were recorded in the Bultfontein area of Free State Province, followed by an explosive epidemic of 548 outbreaks during February–August that spread throughout the central plateau of the country to the southern coastal regions of Western Cape Province. The risk map for December 2009 indicated high risk for the area where the first 2 outbreaks occurred in January 2010 (Figure 8, panel A), and the risk map for January 2010 showed moderate to high risk for the whole central plateau, where outbreaks occurred during February 2010 (Figure 8, panel B). The risk map for February 2010 indicated that risk for outbreaks extended even farther south and that outbreaks occurred during March–July 2010 (Figure 8, panel C). No risk for outbreaks was indicated for the southern parts of the Western Cape Province where 8 outbreaks occurred, although irrigation is practiced fairly commonly in the region.

#### January–July 2011 Outbreaks

**Figure 9:**
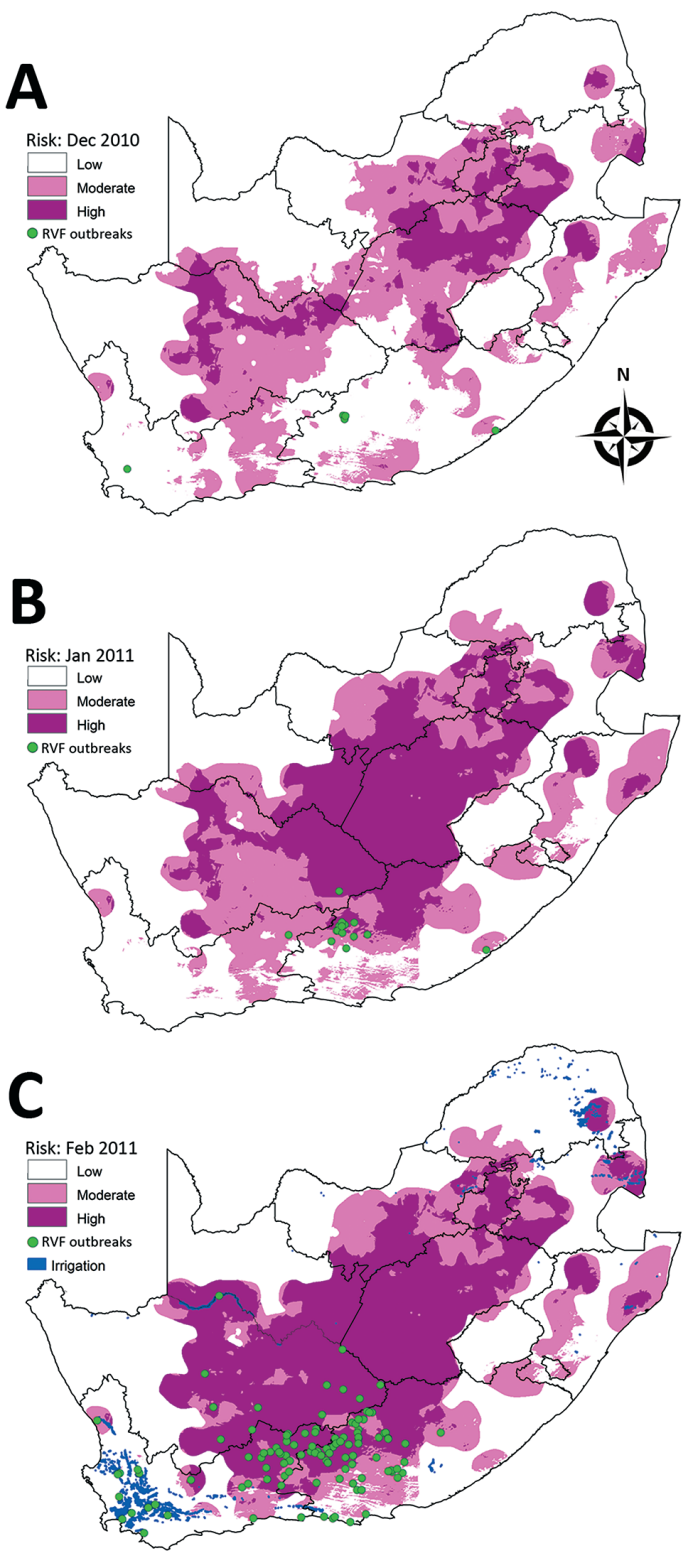
Risk maps for probability of Rift Valley fever (RVF) outbreaks in different areas of South Africa. A) Map for December 2010 showing subsequent outbreaks in January 2011. B) Map for January 2011 showing subsequent outbreaks in February 2011. C) Map for February 2011 indicating irrigation areas and subsequent outbreaks during March–June 2011.

A total of 136 outbreaks were recorded during January–July 2011 in the inland part of the country south of the Orange River, including regions of Northern, Eastern, and Western Cape Provinces. In the risk map for December 2010, no risk was shown for a few areas where outbreaks occurred in January 2011 (Figure 9, panel A), but moderate to high risk was indicated in the risk map of January 2011 for areas of outbreaks in February 2011 (Figure 9, panel B). The risk map for February 2011 showed moderate to high risk for areas of outbreaks during March–June 2011 (Figure 9, panel C). Similar to the previous year, a number of outbreaks occurred in the southern parts of the Western Cape Province where no risk was predicted but where irrigation was practiced.

### Retrospective Evaluation

A total of 778 outbreaks occurred during the epidemics of 2008–2011; of these, 88 (11.3%) were classified by the model as low risk, 236 (30.3%) as moderate risk, and 454 (58.4%) as high risk, indicating that the model correctly identified 88.7% of outbreaks (Table). For the major inland epidemic of January–August 2010, the model correctly identified 95.8% of the outbreaks, compared with 23% of the outbreaks in Northern Cape Province during October–December 2009; this low risk prediction rate for the 2009 outbreaks strengthens the perception that the 2009 epidemic was triggered by irrigation rather than high rainfall (Figure 4, panel B). Irrigation activity was also associated with 8 outbreaks in 2010 (Figure 9, panel C) and 9 outbreaks in 2011 (Figure 5, panel C) in areas of Western Cape Province where the model retrospectively predicted low risk.

**Table:** Summary of predicated risk for all Rift Valley fever outbreaks during the epidemics of 2008–2011, South Africa*

Year, month of outbreak	No. outbreaks	3-mo rolling maximum risk map	Risk for outbreak		
			Low	Moderate	High
2008					
Jan	5	2007 Oct–Dec	NA	5	NA
Feb	10	2007 Oct–Dec	NA	10	NA
Mar	5	2008 Nov–Jan	NA	NA	5
Apr	7	2008 Nov–Jan	1	NA	6
May	4	2008 Nov–Jan	NA	NA	4
Jun	4	2008 Nov–Jan	1	3	NA
2009					
Feb	6	2009 Nov–Jan	NA	6	NA
Mar	4	2009 Nov–Jan	NA	4	NA
Apr	7	2009 Dec–Feb	1	6	NA
May	1	2009 Dec–Feb	NA	1	NA
Jun	1	2009 Dec–Feb	NA	1	NA
Oct	22	2009 Jun–Aug	22	4	NA
Nov	16	2009 Jun–Aug	16	5	NA
Dec	1	2009 Jun–Aug	1	NA	NA
2010					
Jan	2	2009 Oct–Dec	NA	1	1
Feb	99	2010 Nov–Jan	2	15	82
Mar	257	2010 Dec–Feb	4	83	170
Apr	140	2010 Dec–Feb	2	39	99
May	38	2010 Dec–Feb	8	16	14
Jun	10	2010 Dec–Feb	5	3	2
Jul	1	2010 Dec–Feb	1	NA	NA
Aug	2	2010 Dec–Feb	1	NA	1
2011					
Jan	6	2010 Oct–Dec	6	NA	NA
Feb	15	2011 Nov–Jan	1	8	6
Mar	51	2011 Dec–Feb	2	19	30
Apr	47	2011 Dec–Feb	5	14	28
May	14	2011 Dec–Feb	8	1	5
Jun	3	2011 Dec–Feb	1	1	1
Total (%)	778		88 (11.3)	236 (30.3)	454 (58.4)

*Values were extracted from relevant 3-mo rolling maximum risk maps. NA, not applicable.

## Discussion

Although vaccination is the most effective way to protect livestock against RVF outbreaks, it has always been difficult to convince farmers to vaccinate during long interepidemic periods. Vaccine sales have generally been negligible during interepidemic periods, and once epidemics have begun, vaccination has usually been initiated too late and with coverage too limited to avert outbreaks or prevent considerable losses (*1*,*8*). Without a reliable early warning system supported by an effective vaccination strategy, the history of RVF outbreaks in South Africa is bound to repeat itself.

The basis for mapping specific areas with elevated risk for RVF activity during the epidemics of 2008–2011 was the simultaneous occurrence of elevated soil moisture and high rainfall events, which caused flooding of dambos that created suitable habitats for the development of large populations of mosquito vectors and subsequent outbreaks of disease. Our findings show that SSI anomalies that exceed LTMs by an upper threshold of 20%, followed by a sudden high rainfall event, could serve as a reliable risk indicator of imminent RVF outbreaks. Our model correctly identified the risk for an outbreak in nearly 90% of instances >1 months before they occurred. During an epidemic, the initial spread of RVF virus by active vector dispersal is followed by other transmission mechanisms of lower intensity and over longer distances, including the movement of infectious animals and passive vector dispersal (e.g., wind) (*23*). The sites of outbreaks caused by these means of transmission would not necessarily be associated with higher than normal rainfall and could probably explain some of the outbreaks that occurred in areas of low risk. In this regard, irrigation is of particular importance in outbreaks in the Orange River region during October–December 2009 and in outbreaks that occurred in low-risk areas of Western Cape Province in 2010 and 2011. Findings from previous studies strongly suggest that irrigation could create suitable breeding habitats for mosquito vectors and lead to subsequent outbreaks of RVF (*9*,*24*).

The well-documented RVF epidemics of 2008–2012 provided a unique opportunity for investigating the multifactorial nature of the disease in South Africa (*18*,*22*,*23*), and it was possible to retrospectively identify the critical link between soil saturation, rainfall, and RVF outbreaks. However, this novel modeling approach enabled limited scope for comparison with other prediction models, and certain basic assumptions had to be made in the absence of supporting evidence. The model needs prospective validation in future RVF epidemics, including appropriate modification of parameters to enhance performance, and further research is required to identify other possible factors that could improve risk prediction.

Strategic vaccination of susceptible host populations in potential high-risk areas remains the only viable long-term solution to address RVF in South Africa. Essential components of risk management strategy should include regular serologic surveys to evaluate the immune status of livestock populations, an effective immunization protocol backed by adequate strategic stockpiling of vaccine, and a reliable early warning system to identify areas where livestock could be at risk during seasons of high rainfall. The combination of anomalous high soil saturation and rainfall shows promise as a risk indicator for RVF outbreaks, and by incorporating irrigation as an additional element, the accuracy of the prediction model could probably be improved.
